# iParking: An Intelligent Indoor Location-Based Smartphone Parking Service

**DOI:** 10.3390/s121114612

**Published:** 2012-10-31

**Authors:** Jingbin Liu, Ruizhi Chen, Yuwei Chen, Ling Pei, Liang Chen

**Affiliations:** Department of Navigation and Positioning, Finnish Geodetic Institute, Geodeetinrinne 2, 02431 Masala, Finland; E-Mails: ruizhi.chen@fgi.fi (R.C.); yuwei.chen@fgi.fi (Y.C.); ling.pei@fgi.fi (L.P.); liang.chen@fgi.fi (L.C.)

**Keywords:** multi-sensor fusion, indoor LBS, intelligent parking systems, autonomous navigation, connected car, smartphone intelligent services

## Abstract

Indoor positioning technologies have been widely studied with a number of solutions being proposed, yet substantial applications and services are still fairly primitive. Taking advantage of the emerging concept of the connected car, the popularity of smartphones and mobile Internet, and precise indoor locations, this study presents the development of a novel intelligent parking service called *iParking*. With the *iParking* service, multiple parties such as users, parking facilities and service providers are connected through Internet in a distributed architecture. The client software is a light-weight application running on a smartphone, and it works essentially based on a precise indoor positioning solution, which fuses Wireless Local Area Network (WLAN) signals and the measurements of the built-in sensors of the smartphones. The positioning accuracy, availability and reliability of the proposed positioning solution are adequate for facilitating the novel parking service. An *iParking* prototype has been developed and demonstrated in a real parking environment at a shopping mall. The demonstration showed how the *iParking* service could improve the parking experience and increase the efficiency of parking facilities. The *iParking* is a novel service in terms of cost- and energy-efficient solution.

## 1. Introduction

The changes in economic structures and social living structures have increased life style diversity. New information and communication technologies (ICT) accelerate such changes in this process. Living is not restricted any more to only home environments. People travel daily to and from work, spend their time at various points of interest (POI) such as shopping malls and attractions, and acquire goods and services in extensive environments. Therefore, the city ecosystem, including metropolitan office buildings, urban environments, shopping malls, museums and hospitals is now playing important roles in the modern information society. In addition to on-road navigation, that is widely used, an effective parking service is important to improve the experience and efficiency of daily mobility. Although parking technologies have been employed in parking facilities all around the World, it is still common that people have difficulties to park their cars. For example, it is difficult for drivers to find timely vacant parking spaces, and navigation assistance is not available when Global Positioning System (GPS) does not work well. As a consequence, parking difficulties result in unnecessary driving around city center to just look for a parking space. This therefore, on the one hand, causes extra carbon dioxide emissions and deteriorates the environment of the city ecosystem. This is especially true when many people are simultaneously looking for parking places in a downtown area at peak rush hours. On the other hand, it also increases the risk of traffic accidents when drivers have to search for parking spaces while driving. In addition, unpredictable parking situations make it difficult for people to plan their mobility. All of these degrade the modern city ecosystem experience, and have become a critical challenge in the development of future intelligent transportation systems (ITS) [1].

Most parking systems in use automatically detect the arrival and departure of vehicles using various sensors, and can display current parking space occupancy information. With these systems, however, a user could not proactively reserve a parking space. Guidance from the current location to a parking facility, *i.e.*, on-road navigation, is widely used with a Portable Navigation Device (PND) or smartphone, which is equipped with a GPS receiver [2,3]. However, GPS positioning is not available indoors or underground where most urban parking lots are located. As a result, current parking systems cannot provide indoor navigation functionality within a parking lot. Advanced technologies and novel approaches have to be adopted to create a user-friendly parking experience. Smartphone indoor positioning solutions have been studied in recent years [4–8], but such applications are yet to be substantiated. As a latest initiative, the In-Location Alliance was recently launched to promote deployment of location-based indoor services and solutions, and the alliance was formed by 22 Member companies, including NOKIA, Qualcomm, Broadcom, *etc.* [9]. As the smartphone is one of the most used personal mobile devices, it is practical and cost-efficient to utilize a smartphone platform to develop a novel intelligent parking solution.

This paper presents the development of an intelligent smartphone parking service called *iParking*. Based on the existing infrastructure, the *iParking* service uses the features of the accurate indoor positioning capability of a smartphone, and it includes novel accurate indoor location-based functionalities such as indoor parking navigation and intelligent information services. In this paper, the used smartphone indoor positioning solution is introduced in brief, and the *iParking* service is also demonstrated in a real parking facility at a shopping mall. It is concluded that the *iParking* service is a cost- and energy-efficient solution for the end users and an effective solution for service providers and operators of parking facilities.

## 2. Review of Related Works

An intelligent parking system relies physically on the acquisition of parking space occupancy status information. This kind of information can be obtained by various vehicle detection sensors, which are divided into two categories: intrusive and non-intrusive sensors [10]. Intrusive sensors are typically installed in holes on the road surface by invasive installation procedures such as tunneling under the road surfaces or anchoring to the surface of roads. This type of sensors includes active infrared sensors, inductive loops, magnetometers, magneto-resistive sensors, pneumatic road tubes, piezoelectric cables and weigh-in-motion sensors. Non-intrusive sensors encompass microwave radar, passive acoustic array sensors, passive infrared sensors, RFID, ultrasound and video image processing, and they can be installed easily by mounting the device on the ground or the ceiling of a parking lot. The installation and maintenance of non-intrusive sensors do not result in invasive procedures and traffic disruptions, as usually caused by the utilization of intrusive sensors. The specification, strength and weaknesses of various sensor technologies that are currently utilized for vehicle detection were scrutinized by [10–17]. In the parking lot of the Iso-Omena shopping mall, where the *iParking* service was demonstrated, an ultrasound sensor has been installed at each of parking spaces to detect the presence of a vehicle. These sensors and related infrastructure can be used in the development of the *iParking* service, which thus requires no major additional hardware and installation costs.

Many parking systems have been developed around the World for commercial services or trial operations, and they can be divided into five major categories [1]:

1. Parking information systems, including transit information services of different transport means. This type of systems provide information to aid the decision making process of drivers in choosing their transport means to reach their destinations, and help them in finding a vacant parking space within a parking lot. These systems have been implemented in many major cities in Europe, Japan, and the United States.
2. Parking guidance systems. These systems provide guidance toward a free parking space within a parking facility. The availability and location of a free parking space are unknown for users before they arrive at the facility.
3. Parking reservation systems. A parking reservation system enables users in advance to examine the availability and reserve a parking space at a desired parking facility. However, this kind of systems has not been widely adopted as the users of these systems are difficult to find parking spaces they have reserved due to the lack of indoor location capability.
4. Smart payment systems. These systems are implemented to facilitate the payment for parking as conventional payment methods usually cause delay in the payment process. The utilization of smart payment systems also reduces the cost and staffing requirement for payment handling [18–20].
5. Automated parking systems. Automated parking involves the use of computer controlled mechanisms, which operate machines to automatically place vehicles into allocated spaces. This type of parking systems offers a maximum parking space utilization efficiency, and it is advantageous to use them where the size of parking lots is limited. Additionally, automated parking systems indirectly enhance safety for both drivers and vehicles as parking is operated automatically by machines [21]. Nevertheless, these systems require major investment for construction as well as operation.

Next generation intelligent parking services are expected to provide a comprehensive solution, which will have advantages such as a higher parking space utilization efficiency, a smooth and efficient packing process as well as predictable parking cost [22]. It is currently feasible to develop these kinds of novel intelligent parking services as technologies have advanced.

Infrastructure and critical technologies of new-generation intelligent parking systems have been developed significantly in recent years. Mobile Internet and the concept of the connected car have proceeded at a fast pace. Wireless local area networks (WLANs) and Long Term Evolution (LTE) networks enable ubiquitous Internet access with a smartphone. The concept of the connected car is being substantiated by major vehicle manufacturers that are developing related products and services, such as BMW ConnectedDrive [23], Ford SYNC [24] and GM OnStar [25]. The marketing analyst IHS iSuppli has foreseen a significant five-fold market growth in this area from 2010 to 2015 [26].

Indoor mapping is another critical necessary infrastructure to develop new generation intelligent parking systems. A number of companies, such as Aisle411, Micello, PointInside, Google, NOKIA and NAVTEQ, have delivered various indoor map products for a range of points of interest, such as shopping centers and airports, throughout North America, Europe and Japan. In addition to indoor maps, an accurate indoor positioning solution plays an enabling role along with GPS positioning for universal navigation and intelligent information services, which are required in next generation intelligent parking systems.

This paper is the first to develop and demonstrate an indoor location-based smartphone parking service (*iParking*) in a real parking facility of a shopping mall. In particular, the *iParking* system exploits a Hybrid Indoor Positioning Engine (HIPE) smartphone indoor positioning solution with adequate accuracy, reliability and availability, which is presented in this paper.

## 3. Development of the *iParking* Service

The *iParking* application provides a novel intelligent parking service, which is an integrated solution beyond traditional systems as described above. This section first analyzes user new generation intelligent parking service requirements. It then outlines the functional requirements of the *iParking* service, and finally presents the system architecture design and implementation of the *iParking* service.

### 3.1. User Requirements

The *iParking* service aims at creating a smoother and more efficient packing process, enhancing the utilization efficiency of parking spaces and protecting the environment. Related stakeholders include parking users, *i.e.*, drivers, owners and operators of parking facilities, and the environment, such as air quality and traffic conditions that have direct effects on the public. Customers need be able to know the availability of parking spaces in advance and reserve a parking place before their trips. They also require intelligent services for activities after parking, e.g., navigation toward places of interest, providing intelligent information and finding routes backward their own cars after they have completed their activities. Owners and operators of parking facilities expect to improve the parking space utilization efficiency, and as well to provide a pleasant customer-user experience, and promote their business. Urban authorities, who stand for the public, require less unwanted driving to reduce traffic congestion and environmental pollution. As a summary, a new generation intelligent parking service is required to provide all of the stakeholders with the following advantages:

- Efficient and Proactive Mobility Management. An effective parking solution plays a critical role in encouraging the utilization of more efficient transportation means, and it is effective way to alleviate problems such as traffic congestion, roadway costs, pollution emissions, energy consumption and traffic accidents. It is important for users to be able to manage their daily mobility in a proactive way.
- Enhanced Utilization Efficiency and Saved Parking Cost. An effective parking solution enhances the utilization efficiency and also reduces the costs of operators and customers of parking facilities.
- Intelligent Services. Parking is an intermediate stage between driving and the targeted activities of a trip, so intelligent services are required by users at this stage for their activities after parking. Accurate location knowledge makes the services more effective and intelligent.
- Improved Quality of Business. Intelligent parking services improve the visiting experience of customers. As a result, it promotes business quality.
- More Livable Communities. Intelligent parking systems create a more efficient parking process, and accordingly reduce the land consumption of parking facility construction and contaminant emissions during the parking process. As a result, the parking process becomes more environmentally-friendly, and surrounding communities are more livable.

### 3.2. Functional Requirements

Based on these user requirements, an intelligent parking service should provide customers with functionalities for all three stages of a transportation process, including transport planning, driving, and activities after parking. A parking facility monitors the occupancy status of its parking spaces using vehicle detection sensors, and updates online the occupancy status information through a database. In addition, the database system manages the reservation requests of customers and displays on-site the reservation status of each parking space. Mobile Internet makes the service ubiquitously accessible. The *iParking* service currently provides four types of functionalities as follows:

#### 3.2.1. Transport Planning

- *Booking a Space*. Given a time period, reserve a parking space at a facility of interest. When a specific space is reserved, its geographical coordinates are used as the destination of the navigation route.

#### 3.2.2. Driving

- *On-Road Navigation*. Navigation from the current location to the destination parking facility. The built-in GPS receiver of a smartphone is used in this process.
- *Indoor Navigation*. When a user arrives at the destination parking facility and come into a parking facility indoor environment, indoor navigation is launched to navigate the user to the reserved parking space.

The switch between on-road and indoor navigation is done automatically through the proposed HIPE solution.

#### 3.2.3. Location-Based Services

- *Guidance to Points of Interest*. After parking, this function assists users in finding their POI.
- *Location-Based Information Services*. Information is provided to a user based on his location. Accurate location knowledge makes the services more effective.
- *Finding My Car*. This function helps a user to find and get back to the location where his car was parked.

#### 3.2.4. Operations of Parking Facilities

- View the list of all applicable parking facilities. The service provider publishes online a list of all applicable parking facilities where the *iParking* service is operated, and the client can access the list through the Internet.
- Add/remove parking facilities of interest. With this function, a user picks a set of preferred parking facilities. Thus, only the selected parking facilities are included in the client, and use the memory resource of smartphones.

### 3.3. System Architecture and Implementation

**Figure 1.**
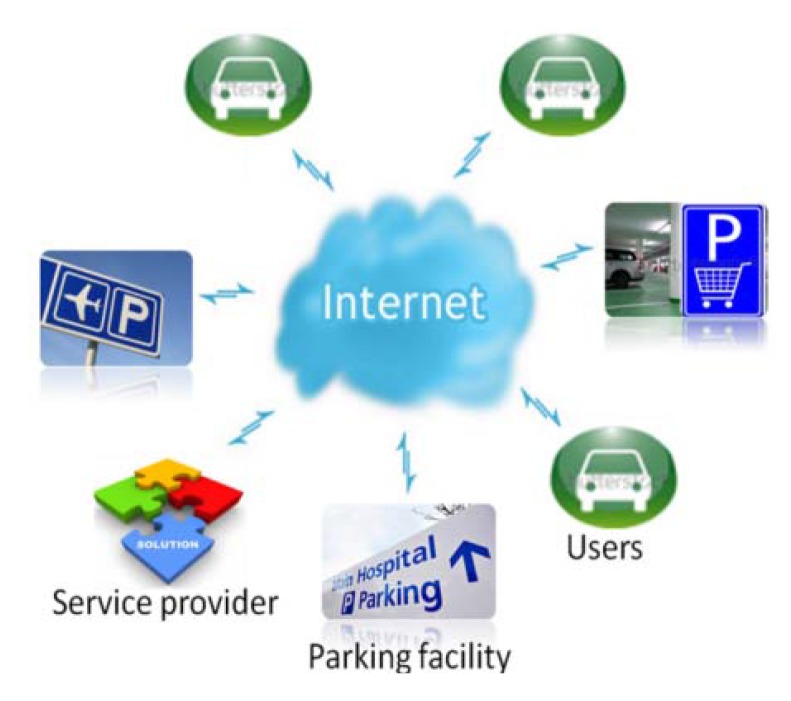
The architecture of the iParking service, which connects parking facilities, users and service providers through the Internet.

The *iParking* service is implemented through a distributed architecture as shown in Figure 1. The Internet connects the different parties, including parking facilities, customers and service providers. The service providers establish the interfaces to bridge parking facilities and users over the Internet, and develop tools to create and maintain the parking facilities database.

Operators of parking facilities use sensors to monitor the occupancy status of each parking space and publish online the parking space occupancy status information in real time. At the same time, the database system handles users' request for parking space reservations, and displays the on-site reservation status to prevent occupancy by others. Every parking facility manages its own database, which consists of information related to the whole parking facility and each of parking spaces. Information related to a whole parking facility includes, for example, permitted parking periods and other general messages, while the information of each parking space includes its geographical coordinates, occupancy status, reservation status, *etc*. At the user end, a client program runs on his smartphone to access the service through the Internet. The client is a light-weight application as a user only collects the databases of the parking facilities of his interest.

**Figure 2.**
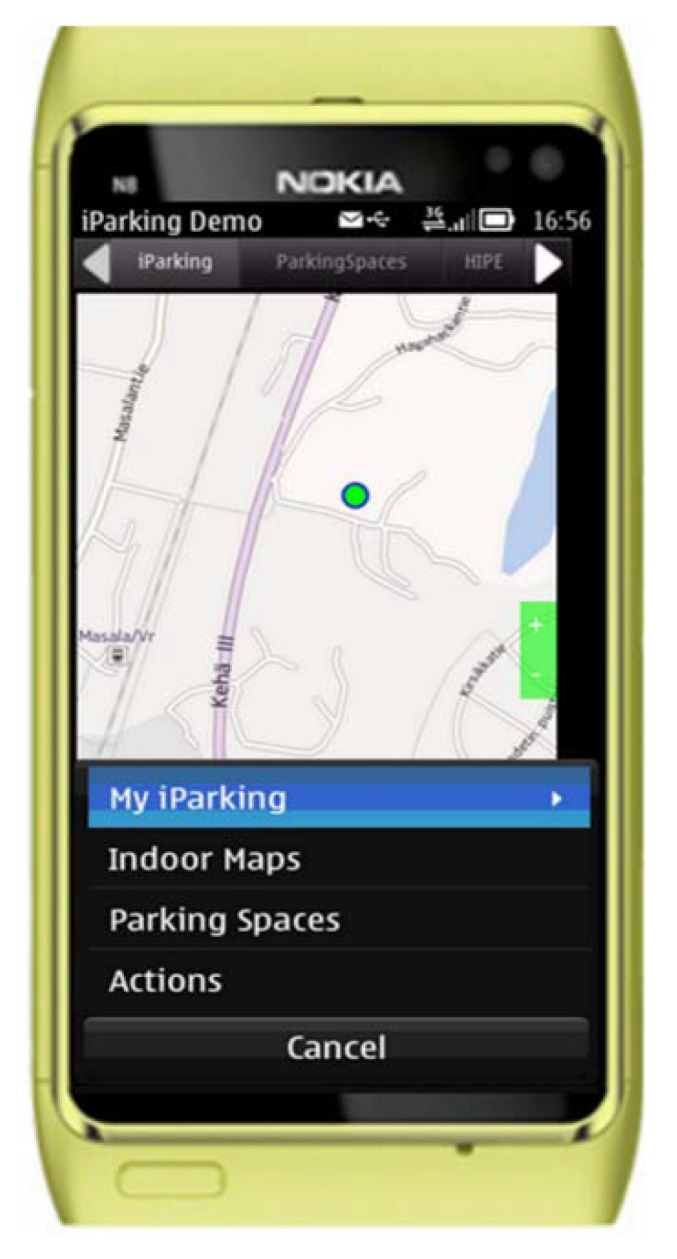
The graphical user interface and main menu of the *iParking* client program, which runs on a smartphone and displays current user position with the flag ( 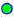) on the map.

User operations of the *iParking* service are conducted on the *iParking* client software, which has a graphical interface as shown in Figure 2. In its current form, the program is developed with a NOKIA N8 smartphone, which runs with Symbianˆ3 operating system (OS). The software development is done with Qt SDK and the Qt Creator integrated development environment (IDE) [27]. QtMobility application programming interfaces (APIs) are used to acquire the measurements of the smartphone sensors for indoor positioning. NOKIA maps are employed for on-road navigation, while an image-based floor map is used as indoor map.

## 4. Smartphone Indoor Positioning Solution

**Figure 3.**
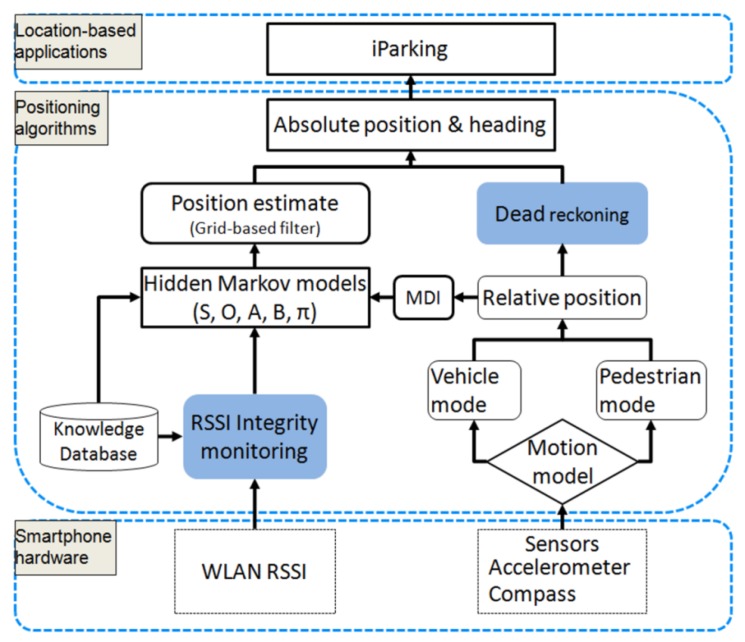
The high-level architecture of the smartphone indoor positioning solution (HIPE), which fuses multiple types of data for indoor location. The blue blocks are new parts that are developed based on the previous works.

As stated earlier in this paper, the *iParking* service exploits an accurate smartphone indoor positioning solution named HIPE. Our previous works [5,28,29] have presented part of the HIPE solution for pedestrian navigation, such as WLAN positioning algorithms, prior knowledge learning, *etc*. In the development of *iParking*, the HIPE solution is extended in two aspects, as shown in Figure 3. First, the integrity of RSSI observables is monitored before they are used for positioning. The integrity monitoring is used to find outlier RSSI observables and exclude them from positioning calculations. Thus, the positioning accuracy is improved. Second, the dead reckoning (DR) technique is utilized to propagate positions during a long interval of RSSI scan, and it enhances the availability of absolute position estimate. This section introduces the operation principle of the HIPE, and evaluates the accuracy and availability of indoor positioning.

The whole HIPE solution, as shown in Figure 3, utilizes the smartphone sensor measurements and WLAN signals from an existing network infrastructure to estimate indoor locations. The methodology of hidden Markov models (HMM) is used to fuse different types of measurements, including RSSI observables and motion dynamics information (MDI) that is measured by the smartphone sensors. The HMM parameters are calculated using the knowledge database of RSSI-location dependency, measured MDI data as well as the result of RSSI integrity monitoring. Together with the HMM and RSSI observables, the grid-based filter, as presented in [6,30], is utilized to estimate absolute positions. Furthermore, between two RSSI observations, relative position is used through the DR approach to propagate absolute locations, and the availability of absolute positions is thus enhanced. The HIPE solution is portable to different smartphone platforms, and configurable to use different combinations of sensors [4].

### 4.1. Indoor Positioning Using WLAN Signals

WLAN RSSI signals have a statistical dependency on geographical locations. The location-RSSI dependency has been widely used for indoor positioning as this kind of positioning solution is cost-efficient and they can be operated in conjunction with communication services. Unlike traditional solutions, which usually use a specific hardware tag for positioning, the proposed HIPE utilizes the built-in hardware of a smartphone to collect the observables and performs position estimation using the smartphone's computational resources. Significant advantages of re-using a smartphone platform include that the positioning solution is more cost-efficient, and it is more convenient to integrate the positioning solution with related applications. However, a disadvantage is that the development of the HIPE is restricted by the API availability of the smartphone platform. For example, the API of Symbianˆ3 operating system used in this paper restricts the interval of two WLAN scans to 8–10 s for energy saving purposes [31]. That means a user will not receive an updated RSSI positioning estimate during such intervals, and this causes an unsatisfactory experience. Therefore, the HIPE propagates locations using the DR technique during the interval of two WLAN scans.

The location-RSSI dependency is learnt and recorded as prior knowledge in a database. Due to the highly non-stationary nature of WLAN signals, RSSI observables at a given location usually have a variance following a non-Gaussian, left-skewed distribution [5]. In this study, a parameteric Weibull function is used to represent the probability density function (PDF) of RSSI observables at a given location, and its three parameters are calculated using a set of RSSI observables [5,31]. It was concluded in [5] that the utilization of the Weibull probability density function can reduce the work load by using a lesser number of RSSI observables than the traditional histogram-based method, and it can improve the positioning accuracy by 20% compared to the histogram-based method when a same number of RSSI observables were used. Given a predetermined grid of reference points (RP), RSSI measurements of multiple WLAN access points (AP) are collected at each RP to calculate the probability distribution of RSSI with the Weibull function. As a result, the learning course produces a database of the probability distributions:

(1) $$\sum =\left\{P\left(O|X=S_{i}\right)\right\}$$

where *P*(*O*|*X* = *S_i_*) is the probability in which a vector of RSSI observables *O* = {*o^k^*}(*k* = 1, ⋯, *M*) is collected at a given RP *S_i_* (*i* = 1, ⋯, *N*), *M* is the number of APs and *N* is the number of RPs.

With the HMM methodology, the HIPE solution considers the motion of a user as a temporally correlated process, instead of a series of isolated points, and it estimates positions using the solutions of HMM problems such as the grid-based filter. The HMM methodology has been presented with details in [6,32], and an implementation of the proposed HIPE has been described in [5,6,33], which included the methods of determining the HMM parameters (*A*,*B*,*π*).

### 4.2. RSSI Integrity Monitoring

The presence of walls, humans and other rigid objects indoors causes multipath and non-line-of-sight propagation of wireless signals, and results in high variance of WLAN RSSI observables that degrades positioning accuracy [34]. Furthermore, a WLAN scanning probably fails to obtain RSSI observables from one or multiple APs due to environmental disturbances or excessive distances between the smartphone and APs. Therefore, it is important to monitor the integrity of RSSIs to improve the positioning accuracy and reliability. In the proposed HIPE solution, two physical natures of RSSI scanning are relied upon in the RSSI integrity monitoring [5]:

Proposition 1: When a scanning mobile device is far away from an access point and its RSSI observable is lower than a threshold, the RSSI variance and the rate of scanning failure are significantly higher.

Proposition 2: The probability distribution pattern of valid RSSI measurements is most matched with that of a given location in the knowledge database when and only when the given location is a true position where RSSIs are observed.

In the proposed HIPE solution, the threshold in Proposition 1 is defined as −75 dBm, and valid RSSI measurements are defined as those observables which are acquired successfully by a scan and are stronger than the threshold. The degree of pattern matching between observed RSSIs and the prior knowledge is numbered by an emission probability, which is a joint probability of all involved measurements and is calculated as follows:

(2) $$b_{i}\left(t\right)=P\left(o_{t}^{1}|X(t)=S_{i}\right)\cdots *P\left(o_{t}^{k}|X(t)=S_{i}\right)\cdots *P\left(o_{t}^{M}|X(t)=S_{i}\right)$$

where *S_i_* stands for *i*-th reference point (*1*≤*i*≤*N*), *t* is epoch number, $o_{t}^{k}$ is the observation of RSSI of *k*-th access point at epoch *t*, $P\left(o_{t}^{k}|X(t)=S_{i}\right)$ is the probability in which RSSI of $o_{t}^{k}$ is observed at a given location *S_i_*, it is obtained from the knowledge database of the location-RSSI dependency.

In the integrity monitoring process, an emission probability is first calculated for each of RPs using RSSIs of all APs, including those of scanning failures, which are marked as “0”. The maximum emission probability and the corresponding RP are recorded as a benchmark. In this case, a scanning failure is considered as useful information that the scanning device is located far away from the corresponding AP with a high probability. Second, valid RSSI measurements are selected to calculate an emission probability for each of RPs. The maximum emission probability of valid RSSI measurements is compared with the benchmark of all RSSIs, and the significance of the difference is evaluated using multiple elements such as the numbers of successful and failing scanning APs respectively, and the numbers of RSSI measurements which have high and low probabilities $P\left(o_{t}^{k}|X(t)=S_{i}\right)$, respectively. If the two sets of emission probabilities are coherent, the emission probabilities of valid RSSI measurements are used to calculate a position estimate. Otherwise, the emission probabilities of all RSSIs are used, and as a result the derived position estimate may suffer major errors with a high probability.

### 4.3. Dead Reckoning with Smartphone Sensors

Relative positions measured by the smartphone sensors are used to propagate positions with the DR technique during a long RSSI scanning interval. In the parking application, relative locations need to be estimated in two motion modes: vehicle mode and pedestrian mode. Vehicle mode is a platform-fixed mode, and it is used for in-vehicle navigation before parking, while pedestrian mode is used for pedestrian navigation and guidance in activities after parking. Different algorithms are used in the different modes. In vehicle mode, the smartphone compass and accelerometers comprise a classic inertial navigation system (INS). Given a fixed body frame, a compass measures motion heading, and measurements of built-in accelerometers are integrated once to produce the velocity, and twice to produce the travelled distance [6]. Consequently, relative location is calculated in a local coordinate system. On the contrary, the integration operation is not applicable in pedestrian mode as it is difficult to keep a smartphone in a fixed body frame. Instead, a pedestrian distance is estimated through accumulating step lengths of occurred steps, which is widely used in pedestrian dead reckoning (PDR) techniques [4,35–38]. Based on the pedestrian acceleration pattern, the occurrences of steps are recognized using accelerometer measurements, and the lengths of all recognized steps are accumulated to calculate moved distance. In this paper, we use a constant value of 0.7 m per step to estimate step lengths. With the estimate of travelled distance (*d_t_*) and heading (*α_t_*) during an interval, relative motion vector *D_t_* is represented in a horizontal plane as follows:

(3) $$D_{t}=\left[\begin{array}{l} d_{t}\cos(\alpha_{t})\\ d_{t}\sin(\alpha_{t}) \end{array}\right]$$

where *t* is an epoch number.

Thereafter, propagated positions in the horizontal plane are calculated based upon a previously determined position as follows [39]:

(4) $$X_{n}=X_{0}+\sum_{t=1}^{n}D_{t}$$

where *t* is an epoch number, *X*_0_ is a previously determined position, *D_t_* is the relative position during a propagation interval. In this study, the propagation interval is 1 s, and position estimate is updated every second through the DR technique during a WLAN scanning interval of 8–10 s. Thus, the availability of positioning solution is enhanced.

### 4.4. Experimental Results

This section presents the experiment results concerning positioning accuracy and availability. In the experiments, we used NovAtel GPS/IMU integrated SPAN system to obtain a trajectory reference. However, as specified in its manual, the SPAN system has increasing errors over time when GPS does not work well indoors. In order to provide the SPAN system periodic references, we recorded nearest true reference points every 30 s during the experiment, and calibrated positioning results of the SPAN system in post mission using the recorded positions of RPs. The calibrated SPAN positioning results achieved decimeter-level accuracy, and were used as the reference of error calculation. Positioning error was calculated epoch by epoch as follows:

(5) $${ɛ}_{i}=\left\|P_{i}-{\bar{P}}_{i}\right\|$$

where *P_i_* is the HIPE positioning result; *P̄_i_* is the reference position.

The experiment lasted for more than 2,000 s, and it obtained 208 RSSI positioning results as the smartphone platform restricts a WLAN scanning interval of 8–10 s. For performance comparison, the recorded RSSI measurements were processed also with the classic Maximum likelihood estimation (MLE) algorithm. The same integrity monitoring processing was applied for the two algorithms, *i.e.*, the grid-based filter and the MLE algorithm. In the HMM approach, the location of the first epoch was estimated using the MLE method, and it was used to calculate the initial probability distribution *π* [6]. The transitional probability matrix *A* was calculated using the measured movement distance and heading, and accordingly the ratio *K* between high and low transitional probabilities adaptively varied at *K* ∈ [2000, 200000] [30]. Given an update interval of 1 s, the dead reckoning approach propagates positions during two scanning epochs based on a previous position estimate of the grid-based filter.

Table 1 compares the positioning errors of the three methods in terms of RMS error (RMSE), average error (AE) and 95% radius error (R95). The grid-based filter has a significantly reduced RMSE by 1.62 m (32.7%), AE by 1.65 m (47.7%) and R95 by 6 m (50%) than the MLE.

**Figure 4.**
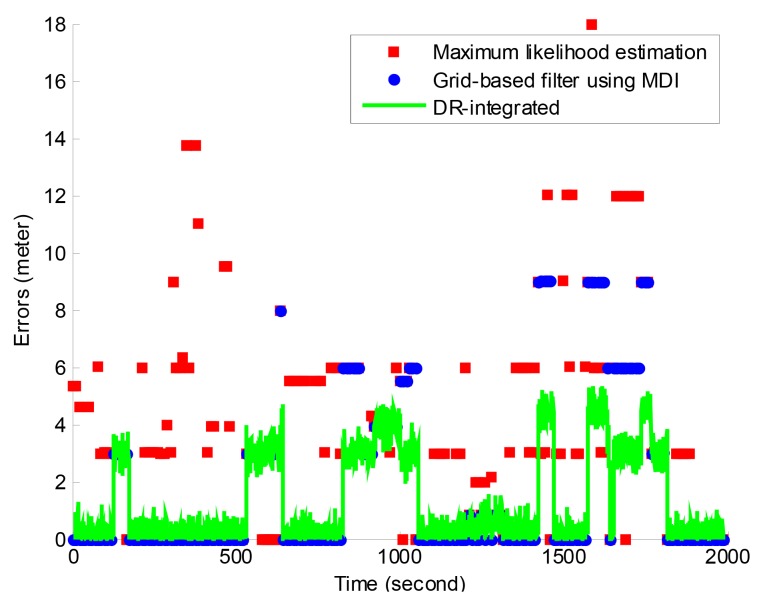
Positioning errors of the three solutions. The DR-integrated solution updated the position estimate at a rate of 1 s, while the grid-based filter and MLE solutions have an update rate of 8–10 s.

Figure 4 shows the time series of positioning errors of the three solutions. It further indicated that the grid-based filter solution has much less errors than the MLE solution, and the DR-integrated solution has an update rate of 1 s, which indicates a higher availability compared to the update rate of 8–10 s in the grid-based filter and MLE solution.

Errors of the DR-integrated solution are dominated by those of the grid-based filter solution as the dead reckoning is based on a latest result of the grid-based filter. However, large errors of the grid-based filter solution are mitigated in the DR-integrated solution as the quality of a new RSSI positioning estimate has been checked using the DR result and large outliers are mitigated in the final DR-integrated solution. The results as shown in Table 1 and Figure 4 indicate the DR-integrated solution, which is the final HIPE solution, has positioning errors of less than the width of a parking space (2.9 m) in most epochs, and it can effectively support the location-based functionalities in the *iParking* service.

## 5. Functional Demonstration of *iParking*

The *iParking* service prototype was demonstrated in a real scenario at the Iso-Omena shopping mall in Espoo, Finland. This section presents the demonstration of the *iParking* service, and shows how the *iParking* service improves the user experience of customers through a virtual story in which a user undertakes a visit for shopping purposes from the office building of the Finnish Geodetic Institute (FGI) to Iso-Omena.

The Iso-Omena shopping mall includes more than 115 stores that offer everything inhabitants and households might need. Meanwhile, the full-service supermarkets contain all the necessary daily goods and groceries. Consequently, a large number of customers visit this shopping mall for daily shopping, and it is not easy to find a parking space at some periods even though there are 2,200 parking spaces in total. An ultrasound sensor has been installed at each parking space to monitor its occupancy status, and the status is published online through the database of the *iParking* service.

**Figure 5.**
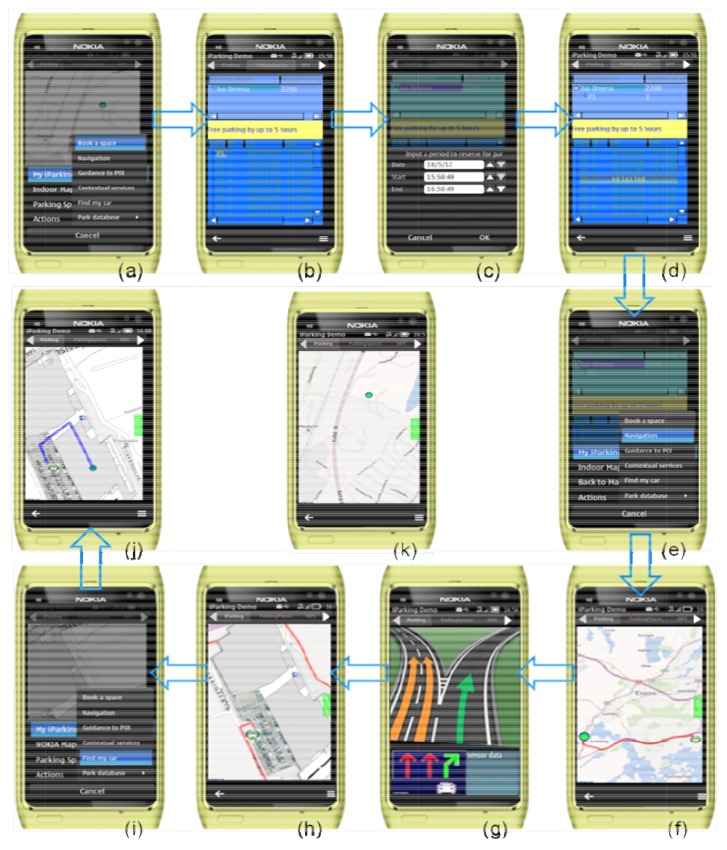
The demonstration of the *iParking* service in the real parking environment of the Iso-Omena shopping mall. The blue arrows indicate the flow of the operation process. Each of the screenshots is explained below.

At the user end, a user runs the *iParking* program on a smartphone, e.g., NOKIA N8 in this paper, which can access the Internet and has built-in NOKIA maps. In the virtual story, the user has retrieved the Iso-Omena *iParking* database on his smartphone as he often visits this shopping mall. Figure 5 demonstrates the whole process of the *iParking* service during the visit. The user reserves a parking space in advance through the *iParking* service before his departure to avoid a parking dilemma. An estimated arrival and departure time are specified to get a list of free spaces for the given period, and the user then selects a preferred space. For example, the user may want to select a space that is the nearest to a particular store he will head for. Once a space is chosen, its coordinates are used as the navigation destination and a departure time and preferable route are recommended. The built-in GPS receiver supports on-road navigation. When the user arrives at the underground park where GPS does not work anymore, the indoor navigation function is launched. The user is guided from his current location to the reserved space. After the user parks his car, the function “Guidance to points of interest” can be used to find the points of interest, e.g., a specific store in this story. As well, related information of various POIs is delivered to the user based on his location. Finally, after the user completes his activities, *i.e.*, shopping in this store, he needs find the route to get back his car.

Then the function “Find my car” navigates the user from his current location to his car.

- A start-up graphic interface and the menu overview. Current position is displayed with the flag ( 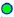) on the map. A click on “Book a space” will open the interface of “Parking places” for related operations.
- The “Parking places” interface, which lists the database of collected parking lots in the above tree widget, and coordinates of all free parking spaces of the selected parking lot at the current moment in the below tree widget.
- A dialog is displayed to enter a specified date and time period to reserve a parking space.
- After the time period of the reservation is specified, the below tree widget is updated to display the list of all free parking spaces at the specified period. A reserved parking space is displayed in the above tree widget.
- A click on “Navigation” starts the calculation of an optimal route and driving time estimate.
- A recommended route is displayed on the map.
- The interface of on-road navigation.
- The interface of navigation inside the parking lot on the ground of an image-based indoor map.
- A click on “Find my car” will display the location of a user's car and an indoor route from his current location to his car.
- A graphical interface that displays the location of a user's car and a route from his current location to his car.
- The main interface of the application, which displays current position with the flag ( 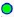) on the map.

In the demonstration of the *iParking* service, we defined the width of a parking space as the tolerable range of navigation errors, and required that a user should be navigated to a correct or adjacent parking space. Based on the operation flow as shown in Figure 5, we have carried out the field demonstration with 14 trials so far. In every trial, a random parking space was booked, and a driver followed the operation process of the *iParking* to perform the visit from FGI to the Iso-Omena shopping center. When the driver completed the visit, he used the function “Find my car” to find the location of the car and the walking route. Thus, each trial included two occurrences of indoor navigation. The visited location in the shopping center was random and different in each trial. We recorded the numbers of parking spaces the *iParking* guided users to at the arrival stage and “Find my car” function, respectively. Navigation errors were defined as offset numbers of parking spaces between correct and navigated parking spaces. Table 2 showed the number of trials that have different offsets between correct and navigated parking spaces.

The width of a parking space is about 2.9 m. The result showed that in almost all cases (more than 85% among our 28 occurrences) a user can find the correct or an adjacent parking space, and in few exceptions he may be navigated incorrectly by up to three parking spaces.

## 6. Conclusions

This paper presented a novel parking service named *iParking*, which features indoor navigation and intelligent services functionalities. The *iParking* service is an environmentally-friendly solution for society, a cost- and energy-efficient solution for end users, and an effective solution for parking operators and service providers.

The proposed indoor positioning solution, which fuses WLAN signals and smartphone sensor measurements, is adequate to enable an intelligent parking service in terms of positioning accuracy, reliability and availability.

A well-managed parking facility can enhance use efficiency and increase the parking revenue. This paper presented the technological feasibility and the effectiveness of a novel parking service based on the indoor positioning solution available in smartphones. The financial aspect was not covered in the paper as it was not considered a technological issue.

Further development of the *iParking* service shall include two aspects. First, more functionalities will be developed to support the operational requirements of the parking operators and intelligent services for activities before and after parking. On the other hand, the service has to be deployed at more parking facilities in order to promote the service to the mainstream market.

## Figures and Tables

**Table 1.** Comparison on positioning errors and availability of the HMM solution, MLE solution and the DR-integrated solution.

**Positioning methods**	**Number of positioning results**	**RMSE (m)**	**AE (m)**	**R95 (m)**
Grid-based filter	208	3.34	1.81	6
DR-integrated solution	2,073	2.06	1.36	5.13
MLE	208	4.96	3.46	12

**Table 2.** The number of trials that have different offsets between guided and correct parking spaces.

**Offsets**	**The number of trials**	

	**Arriving stage**	**Find my car**
0	9	10
1	3	2
2	1	2
3	1	0
